# Association of vitamin D status and cardio-metabolic risk factors in children and adolescents: the CASPIAN-V study

**DOI:** 10.1186/s40795-021-00477-5

**Published:** 2021-11-17

**Authors:** Mostafa Qorbani, Motahar Heidari-Beni, Hanieh-Sadat Ejtahed, Gita Shafiee, Farid Goodarzi, Seyed Saeed Tamehri Zadeh, Majid Khademian, Nami Mohammadian Khonsari, Mohammad Esmaeil Motlagh, Hamid Asayesh, Mohammad Jabbari, Ramin Heshmat, Mehdi Ebrahimi, Roya Kelishadi

**Affiliations:** 1 Non-communicable Diseases Research Center, Alborz University of Medical Sciences, Karaj, Iran; 2 Chronic Diseases Research Center, Endocrinology and Metabolism Population Sciences Institute, Tehran University of Medical Sciences, Tehran, Iran; 3 Child Growth and Development Research Center, Research Institute for Primordial Prevention of Non-Communicable Disease, Isfahan University of Medical Sciences, Isfahan, Iran; 4 Endocrinology and Metabolism Research Center, Endocrinology and Metabolism Clinical Sciences Institute, Tehran University of Medical Sciences, Tehran, Iran; 5 Student Research Committee, Alborz University of Medical Sciences, Karaj, Iran; 6 Department of Pediatrics, Ahvaz Jundishapur University of Medical Sciences, Ahvaz, Iran; 7 Department of Medical Emergencies, Qom University of Medical Sciences, Qom, Iran; 8 Department of Internal Medicine, Faculty of Medicine, Sina Hospital, Tehran University of Medical Sciences, Tehran, Iran

**Keywords:** Vitamin D, Metabolic syndrome, Children, Adolescents

## Abstract

**Background:**

Metabolic syndrome (MetS) starts from early life and is one of the important underlying factors for non-communicable diseases (NCDs) in adulthood. Controversial evidence exists on the role of vitamin D deficiency in increasing the risk of pediatric MetS.

**Objective:**

This study aimed to assess the relationship between vitamin D level with MetS and its components in children and adolescents.

**Methods:**

This nationwide cross-sectional study was performed as part of a surveillance program in Iran. Participants were 2596 students, aged 7 to 18 years, living in 30 provinces. In addition to filling questionnaires, a physical examination was conducted, and blood samples were collected. The serum concentration of 25-hydroxy vitamin D (25(OH)D) was measured using the direct competitive immunoassay chemiluminescence method.

**Results:**

2596 students with a mean age of 12.2 y (55.1% boys) were recruited. Prevalence of vitamin D deficiency and insufficiency in participants was 10.6% (*n* = 276), and 60.5% (*n* = 1570), respectively. The prevalence of MetS was higher in the vitamin D deficient group. Students with deficient vitamin D levels had higher odds of MetS (OR: 4.25, 95%CI: 2.26–7.98), abdominal obesity (OR: 2.24, 95%CI: 1.61–3.12), low HDL-C (OR: 1.65, 95%CI: 1.18–2.30) and high fasting blood sugar (OR: 2.56, 95%CI: 1.43–4.57) in comparison to those with sufficient level of vitamin D.

**Conclusion:**

Vitamin D deficiency was associated with increased odds of MetS and its components in the Iranian pediatric population. These findings underscore the importance of prevention and control of vitamin D deficiency in preventative programs against NCDs.

## Background

Metabolic syndrome (MetS) is a clinical condition characterized by risk factors including dyslipidemia, elevated blood pressure, obesity and impaired glucose regulation. Several factors, including unhealthy diet, physical inactivity and obesity, can affect MetS [1].

Vitamin D levels are generally assessed using the blood concentration of 25-hydroxyvitamin D (25(OH) D). It is essential in the homeostasis of calcium and phosphorus and affects bone mineralization. Recent evidence suggests the extraskeletal effects of vitamin D on cardio-metabolic outcomes. Vitamin D deficiency may increase the risk of non-communicable diseases (NCDs) associated with MetS components [2]. However, there is conflicting and inadequate evidence about the association between vitamin D deficiency and MetS in children and adolescents compared with adults. Some studies showed an inverse association between plasma vitamin D and metabolic syndrome components [3, 4], while others did not reveal any association [5, 6].

Vitamin D deficiency is widespread and is more common in young populations because of sedentary life. In addition, NCDs often begins in childhood or young adulthood [7]. Thus, assessing the link between vitamin D deficiency and cardio-metabolic risk factors in the young is essential. Studies with a large nationally representative sample are needed to clarify conflicting evidence regarding the relationship between plasma vitamin D and the prevalence of metabolic syndrome and its components [8].

Low serum 25(OH)D concentration has been correlated with obesity and MetS; however, few studies have investigated these associations in the Iranian pediatric population, where the prevalence of vitamin D deficiency, obesity, and related metabolic disorders in pediatrics is high. We conducted a cross-sectional study to examine the association between plasma vitamin D status and cardio-metabolic risk factors in a large nationally representative sample of Iranian children and adolescents.

## Methods

### Study population

This cross-sectional study nationwide was performed as part of the fifth survey of a national school-based surveillance program entitled “Childhood and Adolescence Surveillance and Prevention of Adult Non-communicable disease” (CASPIAN-V study). The study was conducted among students aged 7–18 years from primary and secondary schools in urban and rural areas of 30 provinces of the country; Details of the study protocol have been described previously [9]. From 14,400 students who have been enrolled, vitamin D measurement has been done on 2596 students randomly. Written informed consent and verbal assent were obtained from the parents and students after explaining the aim of the study. The study protocol was approved by the Research and Ethics Council of the National Institute for Medical Research Development (NIMAD), and it complied with the declaration of Helsinki.

### Data collection

All students participated in a demographic survey, anthropometric measurements, and blood testing. The demographic survey included questions about the age, region of residence, consumption of vitamin D supplements, family’s socioeconomic status, and leisure time activity. Students’ physical activity (PA) and screen time (ST) during the prior week were assessed using a validated questionnaire. Students were asked about the frequency of their leisure time PA outside of school, which caused sweating or increased heart rate lasting at least 30 min. PA was classified into three groups; low, moderate, and high; defined as having activity 0–2, 3–5, and 6–7 days per week, respectively [10]. ST was considered the average number of hours per day spent watching television, using computers, or playing electronic games. ST was categorized into two groups (low: less than 2 h/d and high: equal or more than 2 h/d) based on international ST recommendations. Questions about the parental educational level and occupational status, school type (public/private), having a private car, and possessing a personal computer was considered in the questionnaire for estimating the family’s socioeconomic status (SES) in three levels (low/moderate/high).

Anthropometric measurements were done according to the standard protocol. Height was measured without shoes. Weight was measured using a digital scale (SECA, Germany) with minimal clothing and without shoes. Body mass index (BMI) was calculated as weight in kg divided by squared height in m^2^. Waist circumference (WC) was measured using a non-elastic tape at the midpoint between the lower margin of the rib cage and the top of the iliac crest to the nearest 0.1 cm. Blood pressure (BP) was measured in a sitting position two times with a five-minute interval using a standardized mercury sphygmomanometer. The first and fifth Korotkoff sounds were considered as systolic blood pressure (SBP) and diastolic blood pressure (DBP), respectively. The average of two measurements was recorded.

Students were asked to fast overnight for 12 h before collecting blood samples. Biochemical variables including fasting blood glucose (FBG), high-density lipoprotein-cholesterol (HDL-C), and triglycerides (TG) were measured by enzymatic methods using Hitachi auto-analyzer (Tokyo, Japan). The serum concentration of 25-hydroxy vitamin D (25(OH)D) was measured using direct competitive immunoassay chemiluminescence method with LIASON 25-OH vitamin D assay TOTAL (DiaSorin, Inc.) according to the manufacturer’s instructions. The coefficient of variation (CV) of this test was 9.8%.

### Definition of terms

MetS was diagnosed based on the Adult Treatment Panel III (ATP III) criteria with modification for the pediatric age group. Students were classified as having MetS if they had three or more of the following components: 1- Serum TG concentration ≥ 150 mg/dl; 2- Serum HDL-C concentration ≤ 40 mg/dL; 3- Serum FBG concentration ≥ 100 mg/dl; 4- Abdominal obesity as waist to height ratio > 0.5; 5- Either SBP or DBP ≥ 90th percentile for age, gender, and height [1]. Vitamin D deficiency is defined as a serum 25(OH)D concentration of less than 10 ng/mL, and vitamin D insufficiency is defined as a serum 25(OH)D concentration between 10 to 30 ng/mL. Serum 25(OH)D level greater than 30 ng/mL was considered as vitamin D sufficiency [11].

### Statistical analysis

Data are presented as mean ± standard deviation or number (percentage). Demographic characteristics and biochemical variables were compared according to gender using independent sample t-test and Chi-square test. The prevalence of MetS and its components was compared according to vitamin D status by Chi-square test. Mean levels of MetS components were compared among vitamin D status groups using Analysis of variance (ANOVA) test. Associations between MetS components and vitamin D levels were examined by linear regression models controlling for potential confounders including age, gender, living area, ST, PA and SES.

Association between vitamin D status and MetS and its components were assessed using multinomial logistic regression analysis. Results are presented as odds ratio (OR) and 95% confidence interval (CI). Three models were defined; Model I: crude, Model II: adjusted for age, gender, living area, and Model III: additionally adjusted for ST, PA and SES. Data analysis was performed using STATA Statistical Software version 11.0 (StataCorp LP. Package, College Station, TX, USA). Significance level was defined as *P*-values below 0.05.

## Results

Participants consisted of 2596 students with a mean (SD) age of 12.2 (3.0) y, including 55.1% boys. The mean of age, WC, SBP and TG, PA level, and family SES were significantly different according to gender (*P* < 0.05). No significant difference existed between boys and girls in terms of place of residence, ST, and level of vitamin D, HDL-C, FBG and DBP. The demographic characteristics of students according to gender are presented in Table 1.

**Table 1:** Participants’ demographic characteristics, according to gender: the CASPIAN-V study

Variables*	Total (*n* = 2596)	Boys (*n* = 1430)	Girls (*n* = 1166)	*P*-value
Age, years^a^	12.18(3.04)	12.32(3.01)	11.99(3.07)	< 0.001
Region^b^				0.79
Urban	1850(71.3)	1022(71.5)	828(71)	
Rural	746(28.7)	408(28.5)	338(29)	
PA^b^				0.01
Low	817(33.4)	417(31.2)	400(36.1)	
Moderate	794(32.5)	435(32.6)	359(32.4)	
High	833(34.1)	483(36.2)	350(31.6)	
ST^b^				0.24
Low	2164(85.5)	1179(84.8)	985(86.4)	
High	367(14.5)	212(15.2)	155(13.6)	
SES^b^				< 0.001
Low	808(32.6)	405(29.4)	403(36.6)	
Moderate	827(33.4)	496(36)	331(30.1)	
High	843(34)	477(34.6)	366(33.3)	
WC, cm^a^	66.75(12.38)	67.31(13.02)	66.05(11.51)	< 0.001
Vitamin D ng/dl^a^	25.94(11.33)	26.11(11.10)	25.74(11.62)	0.40
SBP, mmHg^a^	98.45(12.98)	99.14(13.16)	97.59(12.72)	< 0.001
DBP, mmHg^a^	63.46(10.15)	63.64(10.39)	63.25(9.86)	0.32
HDL-C, mg/dl^a^	46.62(10.12)	46.72(10.24)	46.49(9.96)	0.55
TG, mg/dl^a^	86.45(46.93)	84.86(46.19)	88.41(47.78)	0.05
FBG, mg/dl^a^	91.99 (12.48)	92.35 (13.25)	91.56 (11.45)	0.11

^a^Data are presented as mean (standard deviation)

^b^Data are presented as Number (%)

*BMI* body mass index, *WC*, Waist circumference, *SBP* Systolic blood pressure, *DBP* Diastolic blood pressure, *TC* Total cholesterol, H*DL* High density lipoprotein, *LDL* Low density lipoprotein, *TG* Triglycerides; *FBG* Fasting blood glucose, *PA* Physical activity, *ST* Screen time, *SES* Socioeconomic statue

Prevalence of vitamin D deficiency, insufficiency and sufficiency in students was 10.6% (*n* = 276), 60.5% (*n* = 1570) and 28.9% (*n* = 750), respectively. Prevalence of MetS and its component according to vitamin D status is presented in Table 2. Prevalence of MetS, high FBG, low HDL, high TG and abdominal obesity was higher in vitamin D deficient group compared to vitamin D sufficient and insufficient groups (*P* < 0.01). As shown in Table 3, mean of WC and TG was higher in the vitamin D deficient group compared to two other groups, and HDL concentration was lower in vitamin D deficient and insufficient groups compared to the sufficient group (*P* < 0.001).

**Table 2:** Metabolic syndrome and its component according to vitamin D levels: the CASPIAN-V study

Variable	Vitamin D status			*P*-Value
	Deficient	Insufficient	Sufficient	
Abdominal Obesity	97(35.4)	319(20.3)	147(19.5)	< 0.001
High TG	89(32.5)	379(24.2)	193(25.6)	0.01
Low HDL	94(34.3)	455(29)	168(22.3)	< 0.001
High SBP	5(1.8)	46(2.9)	15(2)	0.29
High DBP	27(9.9)	134(8.5)	83(11)	0.15
High BP	28(10.2)	153(9.8)	86(11.4)	0.47
High FBG	23(8.3)	58(3.7)	26(3.5)	< 0.001
MetS	32(11.7)	48(3.1)	18(2.4)	< 0.001

Abdominal obesity = Waist to height ratio > =0.5; High TG = TG > 100; Low HDL = HDL-C < 40 mg/dl except in boy 15-19y; high systolic BP = SBP at or above the 90th for age, gender and height; high diastolic BP = DBP at or above the 90th for age, gender and height; High BP = either high SBP / DBP at or above the 90th for age, gender and height; Metabolic syndrome = defined according to ATP-III criteria; *SBP* Systolic blood pressure, *DBP* Diastolic blood pressure, *FBG* Fasting blood sugar, *TG* Triglycerides, *HDL* High density cholesterol

**Table 3:** Mean levels of metabolic syndrome components according to vitamin D levels: the CASPIAN-V study

Variable	Vitamin D status	Mean (SD)	*P*-Value
WC	Deficient	69.24(13.29)	< 0.001
	Insufficient	66.80 (12.08)	
	Sufficient	65.73 (12.55)	
SBP	Deficient	98.46(13.10)	0.96
	Insufficient	98.49(12.79)	
	Sufficient	98.34(13.33)	
DBP	Deficient	63.63(9.88)	0.37
	Insufficient	63.24(10.16)	
	Sufficient	63.86(10.23)	
FBG	Deficient	92.90(11.08)	0.43
	Insufficient	91.84(13.60)	
	Sufficient	91.99(10.32)	
TG	Deficient	95.96(56.82)	< 0.001
	Insufficient	85.10(4.86)	
	Sufficient	85.81(46.86)	
HDL	Deficient	45.47(9.20)	< 0.001
	Insufficient	45.77(9.29)	
	Sufficient	48.79(11.64)	

Data are presented as mean (standard deviation)

*SBP* Systolic blood pressure, *DBP* Diastolic blood pressure, *FBG* Fasting blood sugar, *TG* Triglycerides, *HDL* High density cholesterol, *WC* Waist circumference

As can be seen Table 4, the results of linear regression models showed that WC, HDL, and TG were significantly associated with vitamin D deficiency (*P* < 0.001). Moreover, WC and HDL were significantly associated with vitamin D insufficiency (*P* < 0.05). Results of logistic regression analysis investigating the association between vitamin D status and MetS and its components are presented in Table 5. The odds of MetS was higher in the vitamin D deficient group compared with the vitamin D sufficient group; this difference remained significant after adjusting for confounding factors (OR: 4.25, 95%CI: 2.26–7.98). Students with deficient vitamin D levels had a higher odds of abdominal obesity in comparison to those with sufficient vitamin D levels (OR: 2.24, 95%CI: 1.61–3.12). The deficient vitamin D group had higher odds of low HDL-C compared with the sufficient vitamin D group (OR: 1.65, 95%CI: 1.18–2.30). The odds of high FBG in deficient vitamin D group was 2.56 times higher than sufficient vitamin D group (OR: 2.56, 95%CI: 1.43–4.57). In vitamin D insufficient group only the odds of low HDL was higher than sufficient vitamin D group (OR: 1.40, 95%CI: 1.12–1.74).

**Table 4:** Association between metabolic syndrome components and vitamin D levels in linear regression models: the CASPIAN-V study

		Vitamin D status					
		Deficient^1^			Insufficient^1^		
		β	SE	*P*-value	β	SE	*P*-value
WC	Model 1	3.51	0.87	< 0.001	1.06	0.54	0.05
	Model 2	3.44	0.75	< 0.001	1.17	0.47	0.01
	Model 3	3.32	0.81	< 0.001	1.16	0.50	0.02
SBP	Model 1	0.12	0.91	0.89	0.15	0.57	0.78
	Model 2	0.08	0.85	0.92	0.22	0.53	0.66
	Model 3	0.45	0.89	0.61	0.58	0.55	0.29
DBP	Model 1	−0.23	0.71	0.74	−0.62	0.45	0.16
	Model 2	−0.25	0.68	0.70	−0.55	0.43	0.20
	Model 3	−0.00	0.72	0.99	−0.34	0.45	0.44
HDL-C	Model 1	−3.32	0.70	< 0.001	−3.01	0.44	< 0.001
	Model 2	−3.36	0.70	< 0.001	−3.06	0.44	< 0.001
	Model 3	−3.74	0.75	< 0.001	−2.96	0.46	< 0.001
TG	Model 1	10.15	3.30	< 0.001	−0.70	2.07	0.73
	Model 2	9.96	3.30	< 0.001	−0.59	2.07	0.77
	Model 3	9.39	3.42	< 0.001	−0.44	2.12	0.83
FBG	Model 1	0.91	0.88	0.30	−0.14	0.55	0.79
	Model 2	0.89	0.88	0.30	−0.18	0.55	0.74
	Model 3	0.94	0.96	0.32	−0.32	0.59	0.58

^1^In all model sufficient is reference group

Model 1: crude model: Model 2: Adjusted for age, sex and living area; Model 3: additionally adjusted to SES; PA and ST SBP Systolic blood pressure, DBP Diastolic blood pressure, FBS Fasting blood sugar, TG Triglycerides, HDL High density cholesterol, WC Waist circumference

**Table 5:** Association of Vitamin D status with MetS in logistic regression analysis

		Vitamin D status					
		Deficient^1^			Insufficient^1^		
		OR	CI95%	*P*-value	OR	CI95%	*P*-value
MetS	Model 1	4.88	2.708.77	< 0.001	1.21	0.70–2.08	0.48
	Model 2	4.76	2.648.60	< 0.001	1.19	0.69–2.05	0.51
	Model 3	4.25	2.26–7.98	< 0.001	1.17	0.67–2.05	0.56
Abdominal Obesity	Model 1	2.23	1.65–3.03	< 0.001	1.02	0.82–1.28	0.79
	Model 2	2.20	1.62–2.99	< 0.001	1.01	0.81–1.26	1.01
	Model 3	2.24	1.61–3.12	< 0.001	1.03	0.81–1.30	0.79
High SBP	Model 1	.0.67	0.22–2.03	0.48	1.38	0.77–2.46	0.26
	Model 2	0.66	0.22–2.01	0.47	1.35	0.76–2.41	0.30
	Model 3	1.05	0.33–3.34	0.93	1.84	0.94–3.62	0.07
High DBP	Model 1	0.93	0.59–1.46	0.76	0.77	0.58–1.03	0.08
	Model 2	0.93	0.59–1.47	0.77	0.77	0.58–1.04	0.09
	Model 3	1.11	0.68–1.80	0.66	0.83	0.60–1.14	0.26
High BP	Model 1	0.93	0.59–1.45	0.75	0.86	0.65–1.14	0.30
	Model 2	0.93	0.59–1.45	0.76	0.86	0.65–1.14	0.31
	Model 3	1.10	0.68–1.77	0.67	0.92	0.68–1.25	0.60
Low HDL-C	Model 1	1.70	1.26–2.31	< 0.001	1.39	1.13–1.70	< 0.001
	Model 2	1.72	1.26–2.33	< 0.001	1.40	1.14–1.72	< 0.001
	Model 3	1.65	1.18–2.30	< 0.001	1.40	1.12–1.74	< 0.001
High TG	Model 1	1.36	1.01–1.84	0.04	0.90	0.74–1.11	0.34
	Model 2	1.35	1.00–1.82	0.05	0.91	0.74–1.11	0.38
	Model 3	1.28	0.92–1.77	0.13	0.88	0.71–1.09	0.27
High FBS	Model 1	2.53	1.41–4.51	< 0.001	1.06	0.66–1.71	0.78
	Model 2	2.56	1.43–4.57	< 0.001	1.06	0.66–1.71	0.78
	Model 3	1.89	0.96–3.70	0.06	1.10	0.66–1.81	0.70

^1^In all model sufficient is reference group

Model 1: crude model: Model 2: Adjusted for age, sex and living area; Model 3: additionally adjusted to SES; PA and ST

*MetS* Metabolic syndrome, *SBP* Systolic blood pressure, *DBP* Diastolic blood pressure, *FBS* Fasting blood sugar, *TG* Triglycerides, *HDL* High density cholesterol, *WC* Waist circumference

## Discussion

The present study examined the association between plasma vitamin D status and cardio-metabolic risk factors in Iranian children and adolescents. An inverse association was observed between plasma vitamin D and the prevalence of MetS. In addition, plasma vitamin D was inversely related to a number of MetS components. High FBG, low HDL, high TG, and abdominal obesity were higher in the vitamin D deficient group compared to vitamin D sufficient and insufficient groups.

Many cross-sectional studies have documented the relationship between low serum 25(OH) D level and MetS in pediatrics. However, there is inadequate evidence to support a causal link [12]. A study on 6311 US children and adolescents aged 6–18 years showed a potential adverse association between low serum 25(OH) D level and MetS components. However, the underlying mechanisms required further investigation [13].

A study on 3577 US adolescents showed a strong association between low serum 25(OH) D and hypertension, hyperglycemia, MetS, overweight and abdominal obesity [14]. Serum vitamin D levels were inversely associated with FBS, insulin, total cholesterol and TG in the Korean pediatric population [15]. Although local studies on the Iranian population revealed a relationship between vitamin D levels and type 2 diabetes [2, 3] a study on Iranian adolescents demonstrated that vitamin D levels associated with FBS [16] furthermore another study illustrated the relationship between vitamin D levels and obesity in adolescents [5] . Another study on 5867 US adolescents, aged 12–19 y showed an inverse association between serum 25(OH)D and prevalence of MetS phenotype, WC, SBP, and HOMA-IR [17]. Findings on 452 Caucasian children (304 overweight/obese and 148 healthy, normal weight) showed that low 25(OH) D levels were reversely associated with hypertension, total adiposity and MetS [18].

Findings of a systematic review that examine the association between vitamin D status and cardio-metabolic outcomes in generally healthy adults showed positive association between vitamin D insufficiency and disease risk. However, this association was not significant because of the heterogeneity across the studies [19]. Other systematic reviews showed an opposite relationship between vitamin D and cardiovascular risks [20, 21]. In a meta-analysis of 28 studies including 99,745 participants, 43% reduction in cardio-metabolic disorders was associated with the highest levels of serum vitamin D (OR 0.57, 95% CI: 0.48–0.68) [22].

A dose-response meta-analysis on the adult population reported an opposite association between serum 25(OH) D and MetS. This association was shown in cross-sectional studies but not longitudinal studies [23].

Differences in age, sex, country of the studied subjects and mean serum vitamin D levels among participants lead to inconsistency in results [8].

The biological mechanisms by which vitamin D may affect the MetS have not been completely clarified and is complex. Insufficient serum 25(OH) D can change metabolite function and lead to perturbation of many cellular functions including endocrine pancreas. Vitamin D status can be associated with cardio-metabolic risk factors because of the immunomodulatory and anti-inflammatory properties of vitamin D. Vitamin D mediates down-regulation of the production of pro-inflammatory cytokines, stimulate insulin production and improve insulin sensitivity [24, 25]. Vitamin D insufficiency increases C-reactive protein (CRP) level that has been linked to an increased risk of cardiovascular disease, obesity and MetS [26].

In the present study vitamin D deficiency was associated with dyslipidemia. The main component of HDL is apolipoprotein A-1. Vitamin D is essential for maintaining adequate levels of apolipoprotein A-1. In addition, vitamin D is needed for increasing activity of lipoprotein lipase. So, serum vitamin D can be correlated with dyslipidemia [27].

Our results did not show any significant association between low serum vitamin D and blood pressure. Framingham Offspring Study [28] and study on NHANES III data [29] showed an inverse association between 25(OH) D and hypertension or prehypertension. The potential mechanism of these association may be correlated with antihypertensive properties of vitamin D including renoprotective effects, suppression of the renin–angiotensin–aldosterone system, direct effects on vascular cells, and effects on calcium metabolism. However, additional studies are needed before recommendation of widespread vitamin D supplementation in the primary prevention of hypertension especially in pediatric population [30].

Metabolic syndrome is influenced by many factors such as obesity. A review of human, animal, and cellular studies showed inconsistent findings that a low serum vitamin D level was correlated with the cause of obesity [31]. The mechanisms of association between vitamin D deficiency and MetS in obese children should be elucidated in prospective studies and provide health strategies for decreasing the risk of obesity and metabolic syndrome among children and adolescents [32].

The prevalence of vitamin D deficiency among children and adolescents dependents on some factors including ethnicity, sex, physical inactivity, low sun exposure, increased TV watching, low milk intake and high soft drink intake that are also play a role in increased risk of MetS [32, 33]. The association between low serum 25(OH) D and MetS is a concern in pediatric population because children and adolescents with MetS are at an increased risk of future cardiovascular disease and type II diabetes. More studies for the assessment of the effects of vitamin D supplementation on MetS components and prevention of cardiovascular disease is suggested [17].

The strengths of the present study are the large sample size. Some limitations are cross-sectional, lack of imaging procedures and observational design thus we are unable to investigate causality. In addition, some potential confounders might affect our findings.

## Conclusion

The present study demonstrates inverse association between plasma vitamin D level and cardio-metabolic risk factors in children and adolescents. Additional research is essential to support a causal link and determine whether low serum vitamin D levels in childhood may affect the subsequent development of cardiovascular diseases during adulthood. However we should consider vitamin D supplementation in schools, specially in countries with a high prevalence of vitamin D deficiency, as the knowledge on possible benefits of vitamin D and the adverse effects of its insufficiency is groing by day and the beneficial outcomes are to important to ignore.

## Data Availability

The datasets used and analyzed during the current study are available from the corresponding author in response to reasonable requests and with the permission of the patients, for authentication purposes.

## Abbreviations

MetS: Metabolic syndrome
NCDs: Non-communicable disease
25(OH)D: 25-hydroxy vitamin D
PA: Physical activity
ST: Screen time
SES: Socioeconomic status
BMI: Body mass index
FBG: Fasting blood glucose
HDL-C: High-density lipoprotein-cholesterol
TG: Triglycerides
CV: Coefficient of variation
